# Multidimensional School Climate and Mental Health Among Chinese Vocational High School Students: The Role of Personal Growth Initiative

**DOI:** 10.3390/bs16040569

**Published:** 2026-04-09

**Authors:** Yang Cui, Yun Wang, Hongyun Liu

**Affiliations:** 1 State Key Laboratory of Cognitive Neuroscience and Learning, Faculty of Psychology, Beijing Normal University, Beijing 100875, China; cuiyang@mail.bnu.edu.cn; 2 Faculty of Psychology, Beijing Normal University, Beijing 100875, China

**Keywords:** school climate, personal growth initiative, vocational education, mental health

## Abstract

Vocational high school students represent a substantial yet understudied population in school-based mental health research. Drawing on positive psychology and bioecological theory, this study examined whether personal growth initiative (PGI) shows a statistical indirect effect with respect to the relationships between multidimensional school climate and mental health outcomes among Chinese vocational students. Participants were 14,006 students from 112 vocational high schools. Two-level path models simultaneously entered different climate dimensions to estimate their unique associations with PGI, depressive symptoms, and Subjective well-being (SWB) at the within- and between-school levels, controlling for gender and socioeconomic status. Within schools, Safety, Interpersonal Relationships, Rules and Norms/Career Development Support, and Teaching and Learning/Diversity were positively associated with PGI, which in turn was associated with lower depressive symptoms and higher SWB. Wald tests indicated that Safety showed the strongest overall association with depressive symptoms, whereas Interpersonal Relationships showed the strongest overall association with SWB. At the between-school level, school-average climate and school-average PGI were associated with both outcomes, although these findings should be interpreted cautiously given the limited between-school power and substantial overlap among aggregated climate indicators. Overall, the findings are consistent with PGI being an important student-level pathway linking school climate to mental health in vocational education.

## 1. Introduction

Vocational education constitutes a vital component of the global educational system, serving millions of adolescents worldwide. In China alone, vocational high schools enroll approximately 40% of all secondary school students (Ministry of Education, 2024), representing a substantial proportion of the adolescent population. Yet vocational education also differs from general high school education in student composition and educational objectives (Lewis, 1998). In China and other tracked systems, students entering upper-secondary vocational pathways often have lower entrance exam performance and/or constrained family resources, with the vocational track sometimes functioning as a last resort for families seeking earlier work readiness (Guo & Wang, 2020; Hansen & Woronov, 2013).

Students typically enter vocational high schools after completing nine years of compulsory education, usually at around age 15. Compared with general high schools, vocational high schools combine academic coursework with vocational skills training and career preparation, and students often navigate simultaneous demands related to academic adjustment, skill development, identity formation, and future planning. 

A central challenge is that vocational students face pressures that are not only academic. They may also encounter stigma tied to track status, which can affect self-concept and belonging (Alivernini et al., 2020; Allen et al., 2022). Recent evidence from China also suggests that vocational students report substantial levels of stress, anxiety, and depressive symptoms (Su et al., 2024). As vocational students transition from academic-focused general education to employment-oriented training, they directly confront societal realities and encounter psychological challenges related to learning, personal growth, interpersonal relationships, emotional adjustment, and career planning. Compared with general high school students, they may also encounter more setbacks and external pressure in some contexts (Yu, 2022). 

Many also face track-related stigma, lower prior academic confidence, or uncertainty about further education and employment. This distinctive developmental context makes school climate especially important for vocational students’ adjustment, sense of belonging, and mental health. These track-specific experiences highlight the importance of identifying modifiable, school-based protective factors that can support vocational adolescents’ mental health. Despite their demographic significance, vocational students remain underrepresented in educational and psychological research. As they move toward employment-oriented training, they also deal with changes in learning demands, peer relationships, emotional adjustment, and career planning. This raises a practical question for educators and researchers: which school-based factors are most likely to protect students’ mental health in vocational settings? While recent studies have begun addressing this gap (Sun et al., 2024; Symonds et al., 2016), systematic evidence on how school environments shape their psychological adjustment during this critical developmental period remains limited.

Bioecological theory provides a useful starting point. It argues that development is shaped by proximal processes that occur in daily microsystems (Bronfenbrenner, 1979; Bronfenbrenner & Morris, 2007). For vocational students who may have fewer family and community resources, schools become a particularly salient microsystem for daily interaction and support (Downer & Myers, 2010). From this perspective, the school environment is not simply background. It is a key context where psychological adjustment may be shaped.

School climate is commonly defined as the quality and character of school life that captures students’ lived experiences of norms, relationships, and organizational practices (Cohen et al., 2009). It serves as a robust predictor of student well-being across diverse contexts. Importantly, school climate is widely recognized as a multidimensional construct rather than a single undifferentiated environment. Classic syntheses distinguish domains such as safety, relationships, teaching and learning, and institutional or physical environment (Thapa et al., 2013). Empirical evidence demonstrates that these dimensions can show outcome-specific associations. Longitudinal studies reveal that certain dimensions (e.g., school order and teacher respect) prospectively predict lower odds of subsequent health risk behaviors, whereas other domains (e.g., disciplinary style) show distinct patterns for academic outcomes (Ko et al., 2023). Similarly, multilevel analyses indicate differential links between specific climate dimensions and mental health indicators: peer relationship climate is uniquely associated with depressive symptoms, teacher relationship climate is linked with anxiety and hostility, and perceived norms regarding reporting and help-seeking are inversely associated with both internalizing and externalizing symptoms (Franco et al., 2023). These findings suggest that modeling school climate as a multidimensional construct, rather than relying on a global index, is essential for understanding its effects.

For vocational schools, this multidimensional perspective is particularly critical. Vocational education includes skill-oriented instruction, workplace expectations, and potential stigma, which suggests that some climate dimensions may be more psychologically consequential than others. Examining school climate multidimensionally enables the field to move from the general finding that “school climate matters” to the more precise understanding of “which aspects matter most, and why,” thereby generating more targeted intervention implications.

The second gap concerns the mechanism. Even when climate is linked to mental health, the process is often unclear. Accumulating evidence demonstrates that positive school climate is associated with enhanced Subjective well-being (SWB) and life satisfaction while inversely being related to depression and anxiety symptoms (Fernández-Sogorb et al., 2024; Klik et al., 2023; Way et al., 2007). However, school climate effects on mental health are unlikely to operate purely through direct pathways. Emerging mediation and longitudinal evidence suggests that these beneficial effects operate through the cultivation of positive psychological qualities: supportive school environments foster students’ self-efficacy, resilience, and growth-oriented capacities, which in turn buffer against mental health problems and promote psychological well-being (Lera et al., 2023; Llistosella et al., 2023). Personal growth initiative (PGI), defined as individuals’ active and intentional engagement in self-improvement and personal change (Robitschek, 1998), may be a particularly relevant mechanism for vocational adolescents. However, it remains unclear whether PGI can effectively translate environmental supports into mental health benefits within the specific context of vocational education as a positive psychological resource.

To address these gaps, we tested a multilevel mediation model in a large sample of Chinese vocational high school students. We examined seven climate dimensions simultaneously to estimate their unique links with PGI and two mental health outcomes: depressive symptoms and Subjective well-being. This design allows us to move beyond the general claim that school climate matters and instead identify which climate dimensions show the most robust associations and whether PGI helps explain these links.

### 1.1. Literature Review

School climate and students’ mental health. School climate has been defined and assessed in a variety of ways by researchers (Zullig et al., 2010). Nevertheless, a widely accepted definition is provided by Cohen et al. (2009), who characterize school climate as “the quality and character of school life”. School climate represents a multidimensional construct encompassing the quality and character of school life, including interpersonal relationships, institutional norms, teaching and learning practices, physical resources, and organizational structures (Cohen et al., 2009; M.-T. Wang & Degol, 2016).

The relationship between school climate and adolescent mental health has been extensively documented across diverse educational contexts. Positive school climate is robustly associated with enhanced Subjective well-being, life satisfaction, and positive affect while being inversely related to depression, anxiety, and other internalizing symptoms (Aldridge & McChesney, 2018; Fernández-Sogorb et al., 2024; Klik et al., 2023; Varela et al., 2019). When students perceive their school climate as characterized by supportive teacher–student relationships, clear and fair rules, and opportunities for meaningful participation, they develop stronger self-efficacy and more adaptive coping strategies (García-Moya, 2020; Ruus et al., 2007). Conversely, negative school climate features—such as anxiety about aggression and punishment—undermine students’ sense of safety and belonging, increasing their vulnerability to mental health problems (Fernández-Sogorb et al., 2024).

Within vocational education specifically, the relationship between school climate and mental health may be particularly salient. Vocational students often enter these settings having experienced prior academic setbacks or social stigmatization, potentially carrying psychological vulnerabilities (Yu, 2022). For these students, a supportive school climate may serve not only as a buffer against existing distress but also as a catalyst for psychological growth and identity reconstruction. Recent Chinese research suggests that vocational students who perceive supportive and motivational school environments tend to report better mental health outcomes (Fu, 2024; Gao & Meng, 2023). However, while these associations are well-established, the psychological mechanisms through which school climate exerts its effects on mental health outcomes remain incompletely understood, particularly in vocational education contexts where students’ developmental needs may differ from those in general education tracks.

Personal growth initiative and its role in mental health. Personal growth initiative (PGI), conceptualized as an individual’s active and intentional engagement in the process of personal change and development (Robitschek, 1998), represents a positive psychological resource that enables individuals to take proactive steps toward self-improvement. The construct, operationalized through the Personal Growth Initiative Scale-II (Robitschek et al., 2012), comprises four interrelated dimensions: readiness for change, planfulness, using resources, and intentional behavior. This multidimensional conceptualization distinguishes PGI from the passive acceptance of environmental influences, positioning it instead as an agentic, self-directed process of positive development.

A comprehensive meta-analysis revealed that PGI was negatively associated with depression, anxiety, and psychological distress while being positively associated with Subjective well-being, life satisfaction, and positive functioning (Weigold et al., 2020). These effects held across diverse populations and measurement approaches, establishing PGI as a protective factor. More recent research has further demonstrated PGI’s adaptive functions: individuals with higher PGI demonstrate more effective coping strategies and greater self-efficacy in managing life challenges (Weigold et al., 2024).

The theoretical rationale for examining PGI as a mediator between school climate and mental health rests on the premise that environmental support cultivates psychological agency, which in turn drives mental health outcomes. Empirical support for PGI’s mediating role comes from multiple lines of research. Studies have suggested that PGI may function as an indirect pathway linking environmental support to mental health outcomes (Robitschek & Kashubeck, 1999; Robitschek & Keyes, 2009). This mediating role has been supported by prior research in other contexts. For instance, PGI has been found to mediate the relationship between self-compassion and well-being (Arikkatt & Mohanan, 2020), as well as between coping styles and post-traumatic growth (Shigemoto et al., 2017). It may also act as a protective shield to counter the negative effect of higher levels of fear on satisfaction with life (Z. A. Green & Yıldırım, 2022). However, these mediational pathways have been insufficiently examined in vocational education settings. Prior research suggests that a supportive school climate may be especially important for strengthening students’ self-efficacy and willingness to grow. Thus, examining PGI as a mediator in this population addresses both a theoretical gap and a practical concern: theoretically, it clarifies the psychological mechanisms through which school climate influences students’ mental health; practically, it identifies potential intervention points for fostering growth initiative in vocational students.

### 1.2. The Present Study

Despite growing evidence for both the impact of school climate on mental health and the protective role of PGI, several critical questions remain unaddressed. First, studies have linked school climate and PGI to mental health, but we still know little about how they work together. In vocational education settings in particular, whether PGI functions as a process through which school climate influences students’ mental health is rarely tested. Second, many studies examine school climate dimensions one at a time. This makes it difficult to judge which dimensions have unique value once their overlap is taken into account. As a result, we do not yet have a clear answer to a practical question: when multiple climate features co-occur, which ones the most strongly relate to students’ PGI and mental health?

To address these gaps, we tested a multilevel mediation model that included seven school climate dimensions simultaneously. This approach allowed us to estimate the unique contribution of each dimension while controlling for the others. Our goal was to identify the most robust school-based factors linked to students’ growth-oriented agency and mental health. Specifically, we examined PGI as a mediating mechanism connecting multidimensional school climate to both distress, indexed by depressive symptoms, and positive functioning, indexed by Subjective well-being, among vocational high school students.

We hypothesized that (1) multiple school climate dimensions would show unique associations with both PGI and mental health outcomes, (2) PGI would statistically account for part of the association between school climate and mental health, and (3) school climate would function as a multidimensional construct, such that several dimensions would exhibit unique associations with PGI and mental health outcomes even after accounting for overlap among dimensions.

## 2. Materials and Methods

### 2.1. Participants and Procedure 

This study employed a cross-sectional design using a large sample of vocational high school students in China. Data were collected via a secure online survey platform from 112 vocational schools across seven provinces and municipalities in China, including Beijing, Hebei, Shandong, Guangdong and others. These regions spanned the eastern, central, and western parts of China, providing a relatively broad geographic scope, although the sample was not nationally representative because a complex probability sampling procedure was not used. Within each participating school, one class from each grade level (Grades 1 to 3) was selected, and all students in those classes were invited to participate. This study was conducted in accordance with the Declaration of Helsinki and approved by the Ethics Committee of Beijing Normal University (protocol code 527IRB_A_0008_2022001, 25 April 2022). Informed consent was obtained from school administrators, students, and their guardians prior to data collection. The survey protocol strictly adhered to anonymity and confidentiality standards. The online survey system used for data collection required participants to complete all items before submission; accordingly, no item-level missing data were present in the analytic dataset. The final valid sample consisted of 14,006 students (50.4% female, 49.6% male), with a mean age of 16.46 years (SD = 1.18) and an average of approximately 125 students per school. In terms of socioeconomic status (SES), 27.3% of students reported low SES, 62.1% medium SES, and 10.6% high SES.

### 2.2. Measures

School Climate. School climate was assessed using the Vocational School Climate Scale, adapted from established school climate measures (Cohen et al., 2009; Thapa et al., 2013) to better fit the vocational school context in China. The scale comprises seven subdimensions tailored for the Chinese school context: Physical Resources (4 items, e.g., “ The school is well-equipped.”), Safety (4 items, e.g., “Students are safe in the school.”), Rules and Norms (4 items, e.g., “The school rules are fair.”), Teaching and Learning (4 items, e.g., “Teachers explain the course content clearly.”), Interpersonal Relationships (5 items, e.g., “Teachers care about their students.”), Diversity (4 items, e.g., “Different opinions and perspectives are welcomed in our school.”) and a vocational-specific domain, Career Development Support (3 items, e.g., “ The school provides opportunities for students to explore future career paths.”). Participants rated items on a 4-point Likert scale ranging from 0 (completely disagree) to 3 (completely agree). After reverse-coding items where necessary, a composite score was created by averaging the responses across all items of each dimension, with higher scores indicating a more positive perceived school climate. School climate was assessed using a measure adapted specifically for the Chinese vocational high school context. 

The scale was developed on the basis of established school climate instruments used in a large-scale assessment and was subsequently revised to reflect the distinctive characteristics of vocational education in China. Rather than being directly translated word for word from a single existing instrument, the adaptation process prioritized contextual relevance for Chinese vocational high school students. To enhance contextual appropriateness, the initial item pool was reviewed by experts with backgrounds in educational psychology, vocational education, and adolescent development, and a pilot study was conducted to refine item wording and ensure clarity for the target population. Given that the measure was adapted for a specific cultural and educational context rather than directly translated from a source instrument, a formal forward–back translation procedure was not conducted. 

Given that the instrument was adapted for the present context, we conducted a confirmatory factor analysis (CFA) to evaluate its factorial validity. Because all items were rated on a 4-point Likert scale, they were treated as ordered categorical variables, and the CFA was estimated using the WLSMV estimator in Mplus. Given the clustered nature of the data, school-level nesting was accounted for using the COMPLEX option. No residual correlations were specified or freed in the CFA model. The seven-factor model showed acceptable model fit to the data: $\chi^{2}$ = 9658.01; *df* = 356; *p* < 0.001; CFI = 0.945; TLI = 0.937; RMSEA = 0.043; SRMR = 0.025. Standardized factor loadings for all items were robust, ranging from 0.59 to 0.93 (*p* < 0.001), supporting the seven-dimensional structure.

In the present study, Cronbach’s alpha for the subdimensions ranged from 0.851 to 0.926. In addition to the present sample, the adapted school climate scale was also evaluated in an independent regional sample of 6990 vocational high school students. The same general factor structure was supported in that sample, and the reliability estimates were comparable to those observed here, providing supplementary evidence for the structural validity and generalizability of the adapted measure ($\chi^{2}$ = 4415.24; *df* =356; *p* < 0.001; CFI = 0.956; TLI = 0.950; RMSEA = 0.040; SRMR = 0.034). Together with the present findings, these results provide stronger support for the robustness of the adapted scale across samples.

Personal growth initiative. PGI was measured using a previously validated Chinese version of the Personal Growth Initiative Scale-II (PGIS-II; Robitschek et al., 2012). This scale assesses students’ active and intentional engagement in the process of self-change and comprises four dimensions: readiness for change, planfulness, using resources, and intentional behavior. The scale consists of 16 items (e.g., “I figure out what I need to change about myself”), with response options ranging from 0 (Disagree Strongly) to 5 (Agree Strongly). In line with prior research and to reflect students’ overall level of PGI skills, we computed a global PGI score by averaging the four subscale mean scores, yielding a composite score ranging from 0 to 5, with higher values indicating higher PGI. Cronbach’s alpha for this study was 0.967.

Depression. Depressive symptoms were assessed using the Patient Health Questionnaire-9 (PHQ-9; Kroenke et al., 2001). This is a widely validated Chinese version of the PHQ-9, which has demonstrated good reliability and validity in Chinese populations, including adolescent samples (W. Wang et al., 2014). Students reported the frequency of depressive symptoms over the past two weeks. The scale consists of nine items (e.g., “Little interest or pleasure in doing things”), with response options ranging from 0 (Not at all) to 3 (Nearly every day). Item scores were summed to yield a total score ranging from 0 to 27, with higher scores indicating a greater severity of depressive symptoms. Cronbach’s alpha was 0.902.

Subjective well-being. SWB was assessed using the Flourishing Scale (Diener et al., 2010). This 8-item instrument measures psychosocial prosperity, ranging from positive relationships to feelings of competence and meaning. In the present study, items were rated on a 4-point Likert scale ranging from 0 (Strongly disagree) to 3 (Strongly agree). Item scores were averaged to yield an overall flourishing score, with higher scores indicating greater levels of Subjective well-being. In the present study, we used a 4-point response format rather than the original scale format in order to fit the broader survey design for adolescents and to simplify responding in a large school-based assessment. Although this adaptation may reduce direct comparability with studies using the original response format, the scale showed high internal consistency in the present sample. Cronbach’s alpha was 0.928.

Covariates. Demographic variables including gender (0 = male; 1 = female) and socioeconomic status (SES) were included as covariates in all analyses. SES was measured using a subjective social status item: “Compared to your peers, how would you rate your family’s economic status?” The original 5-point responses were recoded into three categories (Low, Medium, High) for analysis because the extreme categories contained relatively few responses. Grade level (Grades 1–3) was recorded during sampling; however, it was not included as a covariate in the final models.

### 2.3. Data Analysis

Data management and preliminary analyses were conducted in SPSS (v26.0), and multilevel path modeling was performed using Mplus (v9.0). First, The Intraclass Correlation Coefficient (ICC) was computed to verify the nested structure of the data (students nested within 112 schools). Second, confirmatory factor analysis (CFA) was performed to validate the measurement structure. Third, because PGI, PHQ, and SWB were modeled as observed composite scores rather than latent variables, we estimated two-level path models using the robust maximum likelihood (MLR) estimator. Models were estimated in Mplus using TYPE = TWOLEVEL to account for the nested structure of the data (students within schools). At the within level, predictor variables were group-mean-centered to disaggregate within-school effects from between-school effects and allowed to covary; gender and SES were specified as covariates predicting PGI and the outcome variables. This centering approach ensures that the within-level coefficients represent the effects of deviations from the school mean. At the between level, school-level aggregates (i.e., school means) were included as predictors to capture between-school effects.

## 3. Results

### 3.1. Common Method Bias Test

Given the cross-sectional nature of the self-report data, the potential influence of common method bias was examined. Following conventional practice, Harman’s single-factor test was conducted as a diagnostic procedure (Harman, 1976; Podsakoff et al., 2003). An exploratory factor analysis including all study items indicated that the first unrotated factor accounted for 34.9% of the total variance, which is below the commonly suggested threshold of 40%. Although common method bias was assessed using Harman’s single-factor test, its potential threat cannot be entirely ruled out. Future studies should therefore consider using multi-informant, longitudinal, or method factor approaches to more rigorously address common method variance.

### 3.2. Preliminary Analyses

The preliminary screening of continuous study variables used in the multilevel path models indicated no severe departures from univariate normality: skewness ranged from −1.07 to 1.44 and kurtosis from −0.81 to 2.16, falling within commonly recommended guidelines (Curran et al., 1996), indicating approximate normality. 

Descriptive statistics and bivariate correlations for all study variables are presented in Table 1. As expected, all dimensions of school climate were positively correlated with personal growth initiative and Subjective well-being and negatively correlated with depression (*p* < 0.001). 

To validate the nested data structure (students nested within schools), Intraclass Correlation Coefficients (ICCs) were computed. The ICCs for the outcome variables were 0.053 for PGI, 0.055 for SWB, and 0.057 for depression, indicate modest clustering. These values support the use of multilevel modeling and suggest that the primary inferences are likely to concern within-school associations.

Prior to model specification, bivariate correlations among all student-level and school-level variables were examined to assess the potential risk of multicollinearity. At the student level, the results indicated that Dimension 3 (Rules and Norms) and Dimension 7 (Career Development Support) were highly correlated, as were Dimension 4 (Teaching and Learning) and Dimension 6 (Diversity), with condition indices exceeding 15 and factor loadings being above 0.5 for both pairs. Given that these dimensions originate from a theoretically established scale comprising seven dimensions, simply deleting items was not a viable approach, as doing so would undermine the content validity and theoretical integrity of the measurement instrument. Accordingly, to address multicollinearity while preserving the full item set, we combined the highly correlated dimensions into composite scores and used their means as observed indicators in the subsequent analysis.

At the school level, correlations among all variables were high (*rs* = 0.587 to 0.934). To mitigate multicollinearity without sacrificing conceptually meaningful content, we adopted a parallel strategy by aggregating all dimensions into a single composite score and using the average as the observed variable at the between level.

### 3.3. The Mediating Role of Personal Growth Initiative

**Figure 1:**
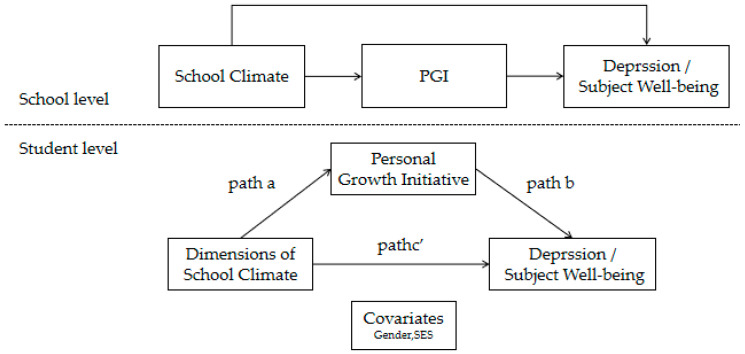
Two-level path model linking multidimensional school climate to mental health via personal growth initiative (PGI).

To examine statistical indirect effects via PGI in the associations between school climate dimensions and mental health, we estimated separate two-level path models for depressive symptoms (PHQ) and Subjective well-being (SWB) among 14,006 vocational high school students nested within 112 schools. Because two pairs of climate dimensions showed high overlap, five within-school climate indicators were entered simultaneously: Physical Resources, Safety, Interpersonal Relationships, Rules and Norms/Career Development Support, and Teaching and Learning/Diversity. Gender and SES were controlled at the within-school level. At the between-school level, an overall school-average climate score was used as the contextual predictor. The analytic model is illustrated in Figure 1. To clarify the estimated structure, this figure distinguishes the within-school model, in which the five climate dimensions were entered simultaneously and allowed to covary, from the between-school model, in which school-average climate was modeled as a composite predictor of school-average PGI and mental health outcomes.

Further results are shown in Table 2 and Table 3.

In the cross-sectional two-level path model predicting depressive symptoms, the within-school paths from school climate dimensions to PGI were estimated first. Physical Resources was not significantly associated with PGI (*b =* 0.019, *p* = 0.397), whereas Safety (*b* = 0.073, *p* < 0.001), Interpersonal Relationships (*b* = 0.287, *p* < 0.001), Rules and Norms + Career Development Support (*b* = 0.133, *p* < 0.001), and Teaching and Learning + Diversity (*b* = 0.347, *p* < 0.001) were all positively associated with PGI. PGI was, in turn, negatively associated with depressive symptoms (*b* = −1.135, *p* < 0.001). After PGI was included in the model, the direct paths from Safety (*b* = −1.124, *p* < 0.001), Interpersonal Relationships (*b* = −0.578, *p* < 0.001), Rules and Norms + Career Development Support (*b* = −0.367, *p* = 0.001), and Teaching and Learning + Diversity (*b* = −0.504, *p* < 0.001) to depressive symptoms remained significant, whereas the direct path from Physical Resources was not significant (*b* = −0.037, *p* = 0.662). The corresponding coefficient products involving PGI were significant for Safety (*b* = −0.083, 95% CI [−0.114, −0.051]), Interpersonal Relationships (*b* = −0.325, 95% CI [−0.382, −0.269]), Rules and Norms + Career Development Support (*b* = −0.151, 95% CI [−0.204, −0.099]), and Teaching and Learning + Diversity (*b* = −0.394, 95% CI [−0.464, −0.323]) but not for Physical Resources (*b* = −0.021, 95% CI [−0.070, 0.028]). At the between-school level, overall school climate was positively associated with PGI (*b* = 1.022, *p* < 0.001), whereas both PGI (*b* = −2.864, *p* < 0.001) and overall school climate (*b* = −1.995, *p* = 0.019) were negatively associated with depressive symptoms. The corresponding coefficient product involving PGI was significant (*b* = −2.926, 95% CI [−4.457, −1.395]), and the total association was also significant (*b* = −4.922, *p* < 0.001).

In the corresponding two-level path model predicting Subjective well-being, the within-school paths from school climate dimensions to PGI showed a similar pattern. PGI was positively associated with Subjective well-being (*b* = 0.198, *p* < 0.001). After PGI was included in the model, the direct path from Physical Resources to Subjective well-being was not significant (*b* = 0.012, *p* = 0.211), whereas the direct paths from Safety (*b* = 0.012, *p* = 0.032), Interpersonal Relationships (*b* = 0.262, *p* < 0.001), Rules and Norms + Career Development Support (*b* = 0.107, *p* < 0.001), and Teaching and Learning + Diversity (*b* = 0.104, *p* < 0.001) remained significant. The corresponding coefficient products involving PGI were significant for Safety (*b* = 0.014, 95% CI [0.009, 0.020]), Interpersonal Relationships (*b* = 0.057, 95% CI [0.047, 0.066]), Rules and Norms + Career Development Support (*b* = 0.026, 95% CI [0.017, 0.036]), and Teaching and Learning + Diversity (*b* = 0.068, 95% CI [0.057, 0.080]) but not for Physical Resources (*b* = 0.004, 95% CI [−0.005, 0.012]). At the between-school level, overall school climate was positively associated with PGI (*b* = 1.021, *p* < 0.001), and both PGI (*b* = 0.434, *p* < 0.001) and overall school climate (*b* = 0.205, *p* < 0.001) were positively associated with Subjective well-being. The corresponding coefficient product involving PGI was significant (*b* = 0.443, 95% CI [0.348, 0.538]), and the total association was also significant (*b* = 0.648, *p* < 0.001). 

Consistent with the modest ICCs (ranging from 0.053 to 0.057), only a small proportion of the total variance in PGI and mental health outcomes was attributable to differences between schools. While the between-school model explained a substantial portion of this limited between-school variance (R^2^_between = 0.673 for PGI and 0.738 for depressive symptoms in the PHQ model; R^2^_between = 0.677 for PGI and 0.962 for Subjective well-being in the SWB model), the absolute amount of between-school variance explained remained small. Therefore, our primary inferences focus on the within-school level, which accounts for the majority of the total variance.

To investigate the relative importance of different paths, a series of Wald tests was conducted to evaluate whether the differences between path coefficients were statistically significant. The Wald test provides a chi-square statistic that assesses the null hypothesis that two or more parameters are equal, allowing for a formal comparison of the relative strength of distinct pathways. 

Wald tests indicated significant heterogeneity across the five dimensions in their total effects on depressive symptoms, *χ*^2^(4) = 128.76, *p* < 0.001, and Subjective well-being, *χ*^2^(4) = 563.15, *p* < 0.001. Safety showed the strongest overall association with depressive symptoms, whereas Interpersonal Relationships showed the strongest overall association with Subjective well-being. In addition, pairwise comparisons of the indirect effects via PGI revealed a broadly similar pattern across the two outcomes: Teaching and Learning/Diversity and Interpersonal Relationships showed the strongest indirect effects, followed by Rules and Norms/Career Development Support and Safety, whereas Physical Resources showed the weakest or non-significant indirect effects. 

## 4. Discussion

### 4.1. Interpretation of Key Findings

Using two-level mediation models, this study examined the unique contributions of five school climate dimensions to personal growth initiative (PGI) and in turn to two mental health outcomes among Chinese vocational high school students. Specifically, Rules and Norms was combined with Career Development Support, and Teaching and Learning was combined with Diversity. We focused on depressive symptoms and Subjective well-being (SWB). All climate dimensions were entered into the models at the same time, so the effects reflect unique associations. Across results, the clearest pattern appeared at the within-school level, which reflects differences between students in the same school.

Hypothesis 1 was supported. First, not all climate dimensions contributed equally to students’ growth-oriented agency. At the within-school level, students who perceived greater Safety, Interpersonal Relationships, Rules and Norms/Career Development Support, and Teaching and Learning/Diversity reported higher PGI. Pairwise Wald tests further suggested that the indirect pathway through PGI was the strongest for Teaching and Learning/Diversity and Interpersonal Relationships, followed by Rules and Norms/Career Development Support and Safety, whereas Physical Resources showed no unique indirect association. In contrast, Physical Resources did not show unique links to PGI once the other climate dimensions were controlled. This pattern suggests that for vocational adolescents, PGI is the most consistently linked to the aspects of the school microsystem that directly structure daily interactions and goal pursuit, specifically clear rules, supportive teaching, and positive relationships. Material conditions may still matter, but their influence may be weaker or overlap with these more immediate experiences.

Second, Hypothesis 2 was also supported. PGI showed a stable association with both dimensions of mental health: higher PGI predicted higher SWB and lower depressive symptoms, which is consistent with earlier work (Ayub & Iqbal, 2012; Luyckx & Robitschek, 2014; Robitschek et al., 2019). Moreover, PGI showed partial mediation at the within-school level. Safety, Interpersonal Relationships, Rules and Norms/Career Development Support, and Teaching and Learning/Diversity were linked to both outcomes through PGI, whereas Physical Resources was not. This detail matters because it suggests that PGI is not simply standing in for a general positive climate perception. Conceptually, examining both depressive symptoms and SWB aligns with the complete state model of mental health, which treats mental illness and positive mental health as related but distinct continua (Keyes, 2005). From this view, PGI can be understood as a growth skill set that supports both fewer symptoms and better functioning (Robitschek & Keyes, 2009). Students with stronger PGI may cope more effectively with challenges and also pursue goals that bring meaning and satisfaction.

These findings are consistent with bioecological accounts of development emphasizing that daily interactions in a person’s immediate setting are central for development (Bronfenbrenner & Morris, 2007). In our study, school climate describes a student’s microsystem, while PGI reflects the student’s capacity to guide and sustain self-change. Supportive climates may foster PGI by providing opportunities, feedback, and modeling that strengthen self-efficacy and goal-directed behavior (Bandura, 2001). It can also support autonomy, competence, and relatedness, which help sustain motivation (Ryan & Deci, 2000). Coupled with evidence that PGI is reliably associated with better mental health across populations (Weigold et al., 2020), our results support PGI as one pathway through which everyday school experiences relate to both higher well-being and lower depressive symptoms.

Hypothesis 3 was partially supported. For SWB, Interpersonal Relationships showed the strongest unique direct links, consistent with the idea that positive affect and life satisfaction are closely tied to feelings of belonging, support, and competence in daily school life (Cohen et al., 2009; Thapa et al., 2013), whereas Rules and Norms/Career Development Support and Teaching and Learning/Diversity formed a second tier with similarly positive direct effects. For depressive symptoms, Safety showed the strongest unique direct association. This finding aligns with the hierarchy of developmental needs, suggesting that perceived safety operates as a proximal protective factor for mental health (M.-T. Wang & Degol, 2016). When students feel physically and emotionally safe, they are less likely to remain on alert and under stress, which can contribute to depressive symptoms (Côté-Lussier & Fitzpatrick, 2016). For vocational students, who may be exposed to higher risks of peer conflict or externalizing behaviors (Bannink et al., 2015; Roorda & Koomen, 2021), safety may be a basic condition for mental stability. Overall, these differences suggest that conclusions depend on which mental health outcome is examined. They also imply that a single global climate score can hide useful details for intervention planning.

Although PGI showed statistically significant indirect associations in several within-school pathways, some direct effects of climate remained significant. This pattern was also reflected in the Wald comparisons, which showed that the relative importance of specific climate dimensions differed depending on whether direct, indirect, or total effects were considered. This suggests that school climate influences mental health through more than one route. This pattern aligns with theoretical models proposing multiple mechanisms by which school environments influence student well-being (Singla et al., 2021). Specifically, safety and coherent rules may reduce psychological distress by directly minimizing exposure to environmental stressors and reducing uncertainty, which have been documented to influence adolescent mental health independent of individual growth resources (K. H. Green et al., 2023; Schweizer et al., 2023).

In addition, the relationship-focused aspects of school climate may promote well-being by strengthening school connectedness, perceived acceptance, and access to social support. These mechanisms are conceptually distinct from PGI, yet they may function as complementary protective pathways for mental health (Núñez-Regueiro & Wang, 2024). These findings are consistent with evidence demonstrating that supportive psychosocial school climates predict reduced health complaints, lower truancy rates, and enhanced help-seeking behaviors under threat conditions (Eliot et al., 2010; Virtanen et al., 2009). Recent multilevel intervention studies are consistent with this view. They suggest that improvements in school climate can benefit adolescent mental health through more than one route. The relational processes and structural features of the school environment may both play a role, and some direct effects often remain even after key mediators are included in the model. Importantly, evidence also indicates that the quality of teacher–student and peer relationships tends to be a stronger predictor of mental health than academic commitment alone (Singla et al., 2021). This aligns with our findings and highlights the practical importance of relational climate in vocational schools.

At the between-school level, school-average climate was associated with school-average PGI and, through PGI, with better school-average mental health. However, these between-school results should be interpreted cautiously. The ICCs were modest, the number of clusters was limited, and the school-level climate indicators were highly overlapping, which constrained dimension-specific contextual inference. Substantively, the present evidence is therefore stronger for within-school associations than for between-school processes. In other words, vocational students’ mental health may depend more on how students within the same school differ in their daily experiences than on average differences between schools. This is consistent with evidence that student-level perceptions of school climate often explain much more variance in mental health than school-level aggregates (Aldridge & McChesney, 2018; Hinze et al., 2024) and that individual students within the same school can experience markedly different psychosocial environments depending on their specific classroom assignments, peer networks, and teacher interactions.

### 4.2. Implications for Intervention and Practice

From a practice perspective, the present findings may help identify two promising areas for future intervention development in vocational education, although the cross-sectional design does not permit causal conclusions. At the environmental level, initiatives that strengthen relational support (e.g., teacher professional development for supportive interactions and classroom attachment) have been shown to promote better adjustment and well-being (Bergin & Bergin, 2009). Similarly, establishing and maintaining fair, transparent, and consistently applied disciplinary systems can reduce uncertainty and promote a sense of safety that directly benefits psychological well-being (Kutsyuruba et al., 2015; Wambua et al., 2024). At the individual level, PGI appears to be a malleable, skill-like resource that can be cultivated through structured training in goal setting, planfulness, and adaptive resource use (Luyckx & Robitschek, 2014). Meta-analytic evidence confirms that PGI can be enhanced through targeted interventions involving psychoeducation, goal-setting activities, and guided practice in planfulness and adaptive resource use (Weigold et al., 2020). Intentional Growth Training (IGT), a brief evidence-based intervention combining PGI education with experiential growth activities, has demonstrated effectiveness in increasing PGI and improving mental health outcomes across diverse populations (Thoen & Robitschek, 2013). The observed within-level associations suggest that, at the student level, intentional growth initiatives may be worth exploring as potential avenues for fostering positive development in vocational high school settings. Because PGI showed consistent indirect links with both depressive symptoms and Subjective well-being, PGI-focused programs may be useful for both the prevention of internalizing distress and the promotion of positive functioning. However, given the cross-sectional design of the present study, causal conclusions cannot be drawn; longitudinal or experimental designs are needed to rigorously evaluate the effectiveness and feasibility of such approaches before implementation. In addition, although validated Chinese versions were used for standardized measures, the adapted school climate scale did not undergo a formal forward–back translation procedure, which may limit cross-cultural comparability.

### 4.3. Limitations and Future Directions

Several limitations of the present study should be acknowledged. First, the cross-sectional design precludes strong causal inferences regarding the directional relationships among school climate, personal growth initiative, and mental health outcomes. The hypothesized model is theory-driven, and the multilevel results are consistent with it, but the direction of effects cannot be confirmed. In addition, the cross-sectional design precludes conclusions about temporal ordering or causal inference. Although we tested indirect effects consistent with our conceptual model, these findings should be interpreted as statistical indirect effects rather than evidence of developmental sequencing or causal mediation. Future studies should use longitudinal or experimental designs to test temporal ordering more directly. For example, prospective data could examine whether changes in school climate are followed by increases in PGI and subsequent improvements in mental health. It would also be valuable to test the reverse pathway, namely whether students with higher PGI come to perceive their school environments more positively over time.

Second, all study variables were assessed via self-report measures, which may introduce shared method variance and reporting biases. Although diagnostic tests suggested that common method bias was unlikely to substantially distort the observed associations, future studies would benefit from incorporating multiple informants or data sources, such as teacher reports, administrative records, or observational assessments of school climate. Such approaches could provide a more comprehensive and objective evaluation of environmental characteristics and student functioning. In addition, the Flourishing Scale was administered using an adapted 4-point response format, which may limit direct comparability with the findings based on the original response options.

Finally, the present study focused on vocational high school students within a specific national and institutional context. Although this focus enhances contextual relevance, it may limit the generalizability of the findings to other educational systems or student populations. Future research should replicate and extend the present model in diverse vocational settings and across cultural contexts. Moreover, intervention-based studies are needed to determine whether school-level efforts aimed at strengthening relational climates, institutional rules, and students’ agency can produce sustained improvements in mental health outcomes. Such work would not only test the robustness of the proposed mechanisms but also inform the design of evidence-based practices tailored to the unique developmental needs of vocational students. 

## 5. Conclusions

This study extends research on vocational students’ mental health by suggesting that personal growth initiative (PGI) may be one student-level pathway linking school climate to both depressive symptoms and Subjective well-being. When the five climate dimensions were considered simultaneously, the most consistent student-level associations emerged for Safety, Interpersonal Relationships, Rules and Norms/Career Development Support, and Teaching and Learning/Diversity. At the level of direct effects, Safety was the most salient for depressive symptoms, whereas Interpersonal Relationships was the most salient for Subjective well-being. These dimensions were indirectly related to mental health through PGI, while several direct effects also remained, suggesting that school climate operates through multiple mechanisms.

The findings also have practical relevance. For vocational students facing major academic and career transitions, schools that foster a sense of safety, fairness, and belonging may provide a stronger foundation for both well-being and reduced distress. Interventions may be especially promising when they combine climate improvement efforts with programs that strengthen students’ goal setting, planning, and adaptive use of resources. At the same time, because the data were cross-sectional and self-reported, these conclusions should be interpreted as evidence of robust associations rather than causal effects. Longitudinal, multi-informant, and intervention-based studies are needed to test whether strengthening these specific climate dimensions and PGI leads to sustained improvements in mental health.

## Figures and Tables

**Table 1:** Means, standard deviation, and correlations for study variables.

Variable	M (SD)	2	3	4	5	6	7	8	9	10
1. Physical Resources	2.18 (0.74)	0.40 ***	0.60 ***	0.54 ***	0.47 ***	0.59 ***	0.58 ***	0.30 ***	−0.26 ***	0.39 ***
2. Safety	1.88 (0.87)	-	0.35 ***	0.43 ***	0.31 ***	0.38 ***	0.31 ***	0.25 ***	−0.34 ***	0.29 ***
3. Rules and Norms	2.36 (0.64)		-	0.53 ***	0.50 ***	0.58 ***	0.83 ***	0.33 ***	−0.28 ***	0.44 ***
4. Teaching and Learning	2.13 (0.66)			-	0.51 ***	0.78 ***	0.50 ***	0.40 ***	−0.32 ***	0.47 ***
5. Interpersonal Relationships	2.30 (0.59)				-	0.52 ***	0.48 ***	0.36 ***	−0.29 ***	0.52 ***
6. Diversity	2.27 (0.68)					-	0.59 ***	0.36 ***	−0.30 ***	0.45 ***
7. Career Development Support	2.40 (0.65)						-	0.31 ***	−0.25 ***	0.42 ***
8. Personal growth initiative (PGI)	3.48 (1.06)							-	−0.38 ***	0.56 ***
9. Depressive Symptoms	4.53 (5.15)								-	−0.47 ***
10. Subjective well-being (SWB)	2.20 (0.60)									-

*** *p* < 0.001.

**Table 2:** Multilevel path model predicting depressive symptoms (PHQ).

Predictor	Path to PGI (a) B (SE) [95% CI]	Direct Effect on PHQ (c′) B (SE) [95% CI]	Indirect Effect via PGI (ab) B (SE) [95% CI]	Total Effect B (SE) [95% CI]
Within-school level				
Physical Resources	0.019 (0.022) [−0.024, 0.062]	−0.037 (0.084) [−0.202, 0.128]	−0.021 (0.025) [−0.070, 0.028]	−0.058 (0.079) [−0.213, 0.098]
Safety	0.073 *** (0.014) [0.046, 0.100]	−1.124 *** (0.065) [−1.253, −0.996]	−0.083 *** (0.016) [−0.114, −0.051]	−1.207 *** (0.067) [−1.338, −1.076]
Interpersonal Relationships	0.287 *** (0.022) [0.243, 0.330]	−0.578 *** (0.089) [−0.752, −0.404]	−0.325 *** (0.029) [−0.382, −0.269]	−0.904 *** (0.085) [−1.070, −0.737]
Rules and Norms + Career Development Support	0.133 *** (0.024) [0.086, 0.181]	−0.367 *** (0.112) [−0.587, −0.147]	−0.151 *** (0.027) [−0.204, −0.099]	−0.518 *** (0.114) [−0.742, −0.295]
Teaching and Learning + Diversity	0.347 *** (0.026) [0.296, 0.397]	−0.504 *** (0.103) [−0.705, −0.303]	−0.394 *** (0.036) [−0.464, −0.323]	−0.897 *** (0.106) [−1.106, −0.688]
PGI → PHQ (b path)	−1.135 (0.051) [−1.235, −1.036]			
Between-school level				
School-average climate (total score)	1.022 *** (0.081) [0.863, 1.180]	−1.995 * (0.849) [−3.660, −0.331]	−2.926 *** (0.781) [−4.457, −1.395]	−4.922 *** (0.415) [−5.735, −4.108]
PGI → PHQ	−2.864 *** (0.681) [−4.199, −1.530]			

Note. Unstandardized coefficients are reported. Within-school predictors were group-mean-centered. Models were adjusted for gender and socioeconomic status at the within-school level. The merged predictors were defined as Rules and Norms + Career Development Support and Teaching and Learning + Diversity. $R^{2}$ (within): PGI = 0.183; PHQ = 0.197. $R^{2}$ (between): PGI = 0.673; PHQ = 0.738. Coefficients whose 95% CI excludes zero may be interpreted as statistically significant. * *p* < 0.05, *** *p* < 0.001.

**Table 3:** Multilevel path model predicting Subjective well-being (SWB).

Predictor	Path to PGI (a) B (SE) [95% CI]	Direct Effect on SWB (c′) B (SE) [95% CI]	Indirect Effect via PGI (ab) B (SE) [95% CI]	Total Effect B (SE) [95% CI]
Within-school level				
Physical Resources	0.019 (0.022) [−0.024, 0.062]	0.012 (0.009) [−0.007, 0.030]	0.004 (0.004) [−0.005, 0.012]	0.015 (0.010) [−0.003, 0.034]
Safety	0.073 *** (0.014) [0.046, 0.099]	0.012 * (0.006) [0.001, 0.023]	0.014 *** (0.003) [0.009, 0.020]	0.027 *** (0.006) [0.015, 0.038]
Interpersonal Relationships	0.287 *** (0.022) [0.243, 0.330]	0.262 *** (0.013) [0.237, 0.287]	0.057 *** (0.005) [0.047, 0.066]	0.319 *** (0.014) [0.292, 0.345]
Rules and Norms + Career Development Support	0.133 *** (0.024) [0.086, 0.181]	0.107 *** (0.013) [0.081, 0.132]	0.026 *** (0.005) [0.017, 0.036]	0.133 *** (0.013) [0.107, 0.159]
Teaching and Learning + Diversity	0.347 *** (0.026) [0.296, 0.397]	0.104 *** (0.011) [0.082, 0.126]	0.068 *** (0.006) [0.057, 0.080]	0.172 *** (0.013) [0.146, 0.198]
PGI → SWB (b path)	0.198 *** (0.006) [0.186, 0.209]			
Between-school level				
School-average climate (total score)	1.022 *** (0.081) [0.863, 1.180]	0.205 *** (0.044) [0.118, 0.292]	0.443 *** (0.048) [0.348, 0.538]	0.648 *** (0.038) [0.573, 0.723]
PGI → SWB	0.434 *** (0.038) [0.359, 0.508]			

Note. Unstandardized coefficients are reported. Within-school predictors were group-mean-centered. Models were adjusted for gender and socioeconomic status at the within-school level. The merged predictors were defined as Rules and Norms + Career Development Support and Teaching and Learning + Diversity. $R^{2}$ (within): PGI = 0.183; SWB = 0.436. $R^{2}$ (between): PGI = 0.677; SWB = 0.962. Coefficients whose 95% CI excludes zero may be interpreted as statistically significant. * *p* < 0.05, *** *p* < 0.001.

## Data Availability

The data presented in this study are available on request from the corresponding author. The data are not publicly available due to privacy.

## Abbreviations

The following abbreviations are used in this manuscript:

PGI	Personal Growth Initiative
SWB	Subjective Well-being
CI	Confidence Interval
N	Sample Size
SD	Standard Deviation
*p*	*p*-value (statistical)
